# The role of Guidance and Planning on Safety of Ophthalmic Practice during the COVID-19 Pandemic

**DOI:** 10.18502/jovr.v15i3.7445

**Published:** 2020-07-29

**Authors:** Saeid Shahraz, Seyed Farzad Mohammadi, Sare Safi

**Affiliations:** ^1^Institute for Clinical Research and Health Policy Studies, Tufts Medical Center, Boston, Massachusetts, USA; ^2^Translational Ophthalmology Research Center, Farabi Eye Hospital, Tehran University of Medical Sciences, Tehran, Iran; ^3^Ophthalmic Epidemiology Research Center, Research Institute for Ophthalmology and Vision Science, Shahid Beheshti University of Medical Sciences, Tehran, Iran

**Figure 1:**
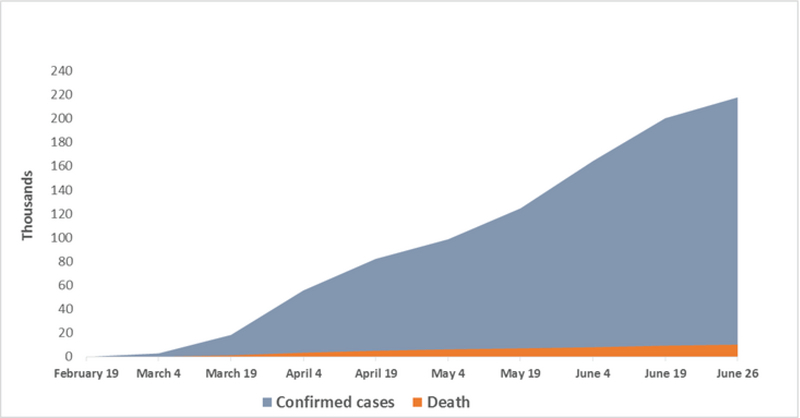
Cumulative number of confirmed cases of COVID-19 and death from February 2020 to June 2020 in Iran.

Coronavirus Disease 2019 (COVID-19) caused by the severe acute respiratory syndrome coronavirus 2 (SARS-CoV-2) was reported for the first time in China in December 2019.^[1]^ It has been spreading rapidly worldwide and has emerged as the most massive health crisis since World War II.^[2,3]^ The World Health Organization (WHO) declared the SARS-CoV-2 outbreak as a pandemic on March 12, 2019.^[4]^ The COVID-19 pandemic is not only a health crisis but also has a significant impact on societies, economies, and progress rates toward the United Nations' sustainable development goals.^[5]^ The Eastern Mediterranean Region (EMR) ranks third in the world in terms of the total number of confirmed cases among the six WHO regions.^[6]^ Iran, as one of the EMR countries, has reported 217,724 confirmed cases and 10,239 deaths from February 19 to June 26, 2020 (Figure 1).^[2]^

A recently published meta-analysis reported that the risk of transmission is reduced by more than 75% through implementing three strategies by both healthcare workers and communities: at least a 1-m social distancing, use of a face masks (surgical or similar masks (12–16-layer cotton or gauze masks), N95 respirators or similar), and eye protection.^[7]^ Recently, the WHO has released interim guidance on the use of masks for avoiding transmission of COVID-19.^[8]^

Infected droplets can find their way into the ocular surfaces where the virus can replicate.^[9]^ An overall pooled prevalence of ocular manifestations in patients with COVID-19 was 5.5% in one report.^[10]^ There is limited published evidence on the prevalence of ocular manifestations of COVID-19 in Iran.^[11]^ In a case series including 43 patients in Iran, reverse transcription-polymerase chain reaction (RT-PCR) on nasopharyngeal and tear samples indicated presence of viral material in 30% and 7% of cases, respectively. The nasopharyngeal RT-PCR results were indicative of the presence of the virus in all patients with positive tear RT-PCR results, which comprised a case with clinical conjunctivitis.^[11]^ Although the rate of ocular manifestations is low, protecting against viral transmission is vital in ophthalmology practice due to the proximity between the examiner and patients, the lengthy period of exposure during examinations, and direct contact with patient's eye secretions.^[12]^ Many non-urgent ophthalmic services were ceased at the ophthalmology centers in Iran, similar to other countries, during the early months of the pandemic to tackle the spread of COVID-19.

Government agencies, ophthalmology societies, and eye health centers have developed guidance and policy statements to reduce the risk of transmission on one hand and maintain continuity of eye care on the other.^[13,14,15,16,17,18,19]^ These blueprints have included recommendations for modifying the clinical pathway and disinfecting protocols, initial screening, and necessary protective equipment for eye care providers, including ophthalmologists, optometrists, clinic/hospital managers, and staff. Furthermore, triage for ophthalmic disorders and cessation of elective examinations, diagnostic procedures, and surgeries were addressed in the guidance.^[13,14,15,16,17,18]^ In response to the COVID-19 pandemic in Iran, the Knowledge Management Unit, Ophthalmic Research Center, Research Institute for Ophthalmology and Vision Science, Shahid Beheshti University of Medical Sciences, in collaboration with the Iranian Society of Ophthalmology published a joint guidance in March 2020, which appears in this issue of the journal.^[20]^ The ad hoc committee urged ophthalmologists to restrict non-emergent services and provide care for conditions such as retinal detachment, trauma, chemical burns, dangerously elevated intraocular pressure, and severe ocular infections. It recommended postponing elective surgeries and counsel patients using remote approaches. The use of face masks, goggles, gloves, and slit-lamp shields were emphasized for visiting urgent cases. Infection control strategies, including handwashing, disinfecting ophthalmic examination equipment with 70% alcohol, and surfaces with bleach-based disinfectants were also recommended.^[20]^

After leaving behind the first peak of the COVID-19 infection, ophthalmology centers were reopened, and eye care services resumed to prevent sight-threatening eye disorders and to manage the backlog of postponed appointments.^[21]^ New guidance was developed for the post-peak era, and existing recommendations were updated. Naveed et al prepared guidance for modifying the ophthalmic workplace for the post-peak period.^[22]^ The post-peak recommendation addressed different strategies, including engineering and administrative controls and protecting workers with personal protective equipment. The authors suggested tele-triage by a senior physician for urgent cases, minimizing face-to-face time in routine clinics, and limiting general anesthesia to absolutely necessary surgical cases.^[22]^ The joint guidance was also updated to provide recommendations for protecting ophthalmic health workers and ophthalmology centers during the post-peak era. It now mandates physical distancing as well as requirements for protecting ophthalmologists and staff during surgeries. Cataract surgery was considered as a semi-urgent operation that could be performed in cases prone to falls and subjects with significantly impaired vision.^[20]^ Eye Health and Prevention of Blindness Office of the Center for Non-communicable Diseases Control of the Ministry of Health and Medical Education issued an official point of view in late June. It offered detailed guidance for the public and health workers, in addition to eye health professionals during the COVID-19 pandemic. It addresses ocular involvement by SARS-CoV-2 as well.

The WHO Regional Director for the EMR notified a risk of an accelerated trend in the number of confirmed cases in EMR countries as a consequence of easing restrictions.^[23]^ Estimations by the Institute for Health Metrics and Evaluation (IHME) also confirmed this trend.^[24]^ Therefore, to continue providing care for ophthalmic patients, it is crucial that ophthalmic care providers and staff follow the guidance strictly to reduce the risk of infection transmission from patients to medical care and vice versa.

In summary, it seems that initial screening at the time of admission, mandating social distancing, washing hands, wearing face masks by healthcare providers and patients, use of slit lamp shields, and disinfecting the instruments are reasonable protective strategies recommended by various guidance. We have to consciously monitor the pandemic status, public health developments, and the evidence which emerges to tailor and update our control measures and eye care strategies. And we all know that the COVID-19 pandemic, directly and indirectly, has affected medical care and will continue to transform it further in the coming decade.
